# Prepulse inhibition of the blink reflex in functional neurological disorder and fibromyalgia

**DOI:** 10.1093/brain/awaf437

**Published:** 2025-11-21

**Authors:** Lucia Nováková, Petr Sojka, David Voženílek, Tomáš Sieger, Lenka Hasíková, Ladislav Šenolt, Jakub Závada, Mark J Edwards, Tereza Serranová

**Affiliations:** Department of Neurology, Charles University in Prague, 1st Faculty of Medicine and General University Hospital in Prague, Prague 128 08, Czech Republic; Department of Neurology, Charles University in Prague, 1st Faculty of Medicine and General University Hospital in Prague, Prague 128 08, Czech Republic; Central European Institute of Technology, Masaryk University, Brno 601 77, Czech Republic; Department of Neurology, Charles University in Prague, 1st Faculty of Medicine and General University Hospital in Prague, Prague 128 08, Czech Republic; Department of Cybernetics, Faculty of Electrical Engineering, Czech Technical University in Prague, Prague 128 00, Czech Republic; Institute of Rheumatology, Prague 128 00, Czech Republic; Institute of Rheumatology, Prague 128 00, Czech Republic; Institute of Rheumatology, Prague 128 00, Czech Republic; Institute of Psychiatry, Psychology and Neuroscience at King’s College London, London SE5 8AB, UK; Department of Neurology, Charles University in Prague, 1st Faculty of Medicine and General University Hospital in Prague, Prague 128 08, Czech Republic

**Keywords:** functional movement disorder, fibromyalgia, prepulse inhibition, pain, sensory processing

## Abstract

Prepulse inhibition reflects subcortical sensory integration, where a low-intensity peripheral stimulus (prepulse) reduces the amplitude of a reflex response to a subsequent high-intensity stimulus. As a measure of pre-attentive sensory gating, prepulse inhibition has been found to be altered in small cohorts of patients with functional disorders, including functional motor disorder and fibromyalgia, suggesting a shared deficit in sensory information processing. However, prior studies have not demonstrated consistent associations between prepulse inhibition abnormalities and clinical measures.

We hypothesized that widespread pain and somatic symptoms in somatic symptom disorders may result from a general deficit in the interpretation of bodily signals, potentially linked to abnormalities in sensory filtering as measured by prepulse inhibition.

In this study, we examined 140 participants across four age- and sex-matched groups: 35 patients clinically categorized with functional motor disorder without fibromyalgia, 35 with both functional motor disorder and fibromyalgia, 35 with fibromyalgia only and 35 healthy controls. A weak electrical stimulus to the index finger served as the prepulse, delivered 100 ms before supraorbital nerve stimulation to elicit the R2 component of the blink reflex. Prepulse inhibition was calculated as the per cent reduction in R2 amplitude.

Across all groups, lower prepulse was significantly associated with higher scores on the Fibromyalgia Severity Scale, consisting of the Widespread Pain Index and Symptom Severity Scale. In patients with functional motor disorder, no association was found between prepulse inhibition size and objectively rated motor symptom severity.

These findings suggest that impaired early sensory processing at the subcortical level is related to ‘fibromyalgianess’ in people with functional motor disorder and fibromyalgia. Abnormal prepulse inhibition may serve as an objective transdiagnostic marker of fibromyalgia symptomatology or fibromyalgianess, including widespread pain and other non-motor symptoms in functional disorders, highlighting a potential role of sensory gating deficits in the pathophysiology of fibromyalgia-spectrum manifestations.

## Teaser

Nováková *et al.* identified altered sensory signal processing linked to chronic widespread pain in people with fibromyalgia and functional motor disorder. Reduced prepulse inhibition was associated with higher fibromyalgia scores, suggesting potential as a transdiagnostic marker of fibromyalgia symptomatology or ‘fibromyalgia-ness’.

## Introduction

**Figure 1:**
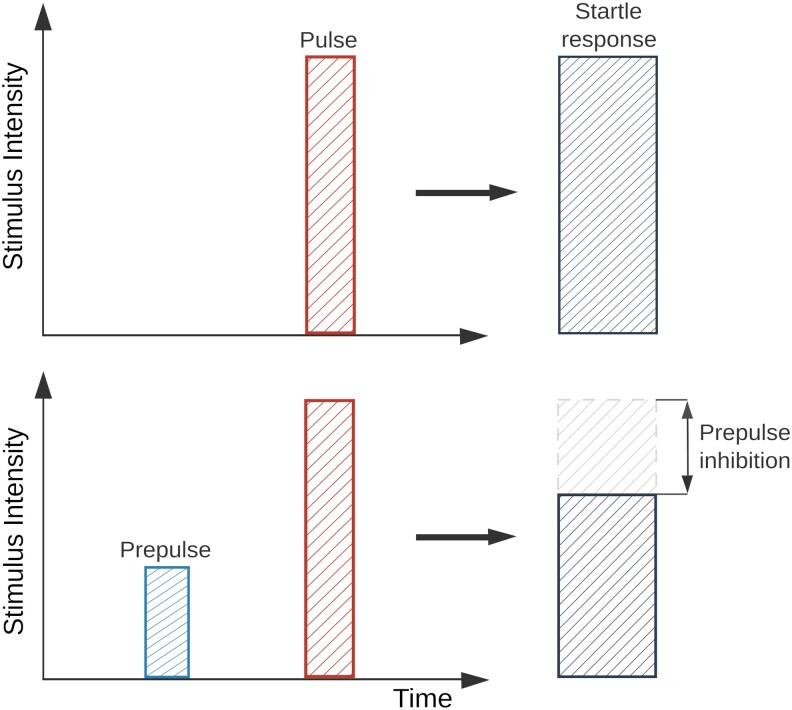
**Prepulse inhibition scheme.** Prepulse inhibition is a neurophysiological phenomenon in which a reflexive response (e.g. the startle response or the blink reflex) to a strong stimulus (pulse) is significantly reduced when preceded by a weaker stimulus (prepulse) within 30 to 500 ms. The prepulse alone is too weak to elicit a reflex but effectively modulates the subsequent response. Prepulse inhibition serves as a key measure of sensorimotor gating, a fundamental mechanism that regulates sensory input by filtering out irrelevant or disruptive signals.^33^

Integration of competing sensory inputs is crucial: it helps prevent sensory overload and allows more effective processing of relevant information.^1^ Prepulse Inhibition (PPI) is a robust neurophysiological phenomenon in which a sensory stimulus (the prepulse) too weak to trigger a reflex on its own, reduces the intensity of a reflex response to a stronger stimulus presented 30 to 500 ms later (Fig. 1). PPI is widely accepted as a key measure of sensorimotor gating, a physiological mechanism that regulates sensory input by integration of competing stimuli.^2^ The top-down regulation of PPI appears to have a critical relationship with the cortico-striatal-pallidal-thalamic network (or more broadly forebrain) input to the brainstem.^2^

PPI has been found consistently reduced in people with schizophrenia, obsessive-compulsive disorder, anxiety disorders, post-traumatic stress disorder, Huntington’s disease and Tourette syndrome.^3^ Interestingly, PPI has also been found abnormal in two major subtypes of functional neurological disorders (FNDs): functional seizures and functional motor disorders (FMD)^4,5^ as well as in somatic symptom disorders with pain such as fibromyalgia,^6^ interstitial cystitis^7^ and irritable bowel syndrome.^8^ While the primary symptoms of FND relate to motor disorders, seizures and sensory loss, pain is also commonly present in people with FND and is typically rated by patients as a major contributor to disability and impaired quality of life.^9^ Chronic pain conditions including fibromyalgia, which is characterized by widespread pain seem to be more frequent in people with FND.^10^ FND and fibromyalgia share common non-motor symptoms, including fatigue, cognitive issues, sleep disturbances and headaches.^11-13^ FND and other somatic symptom disorders are associated with alterations in the functioning of brain networks and are now believed to share underlying pathophysiological mechanisms. Recent theoretical models based on predictive coding accounts of brain function suggest functional symptoms arise from the development of abnormal predictions about motor and sensory states including pain, driven by an abnormal allocation of attention.^14^ People with FMD also exhibit poor sensory discrimination, compatible with underweighting of incoming sensory evidence, especially if the inputs are noisy.^15,16^

Deficient PPI found in people with different functional symptoms/syndromes might suggest that an impairment in ability to integrate sensory inputs could be a common neural mechanism operating at subcortical level under control of top-down forebrain influences. A lack of sufficient filtering or integration could result in increased sensory noise, which in turn could more easily be overridden by prior expectations.^5^

In studies examining PPI in people with functional symptoms (FND and somatic symptom disorders) only a limited number have assessed the relationship between PPI deficits and clinical measures.^4,5^ Notably, these studies have not found significant correlations between PPI abnormalities and symptom severity. For instance, research on patients with FMD reported no significant association between motor and non-motor symptom measures (including pain intensity) and PPI size.

Recent advances in fibromyalgia research have led to revision of diagnostic criteria and to the development of a diagnostic self-reported measure of pain widespreadness and number and severity of other non-motor symptoms such as fatigue, poor sleep, mood and cognitive problems.^12,17^ Fibromyalgia is increasingly viewed not as a discrete disease but as a spectrum of ‘fibromyalgianess’, defined by pain widespreadness and symptom severity scores, and extending to individuals with symptoms who do not meet full diagnostic criteria across clinical and non-clinical populations.^18^ We hypothesized that widespread pain and perceived symptom burdens could result from a general deficit in misinterpretation of bodily signals in people diagnosed with fibromyalgia and FND linked to insufficient filtering of sensory information as measured by PPI.

The aim of this study was to perform a comprehensive investigation of PPI in fibromyalgia and FND, with a particular focus on the relationship between PPI abnormalities and symptom severity rather than on differences between groups with different categorical diagnoses. To achieve this, we studied the association of PPI magnitude and fibromyalgia-related symptom severity on a continuum of fibromyalgianess in three groups of patients with clinically diagnosed FMD and/or fibromyalgia together with age- and sex-matched healthy individuals. In addition, the association between PPI size and objectively rated motor symptom severity was studied in patients with FMD, both with and without comorbid fibromyalgia.

## Materials and methods

One hundred and forty subjects were recruited to the study between January 2022 and March 2024. Exclusion criteria for both the control and patient groups included being under 18 years old, language difficulties, severe learning disabilities or cognitive impairment, significant illnesses associated with non-motor symptoms, substance dependence, psychosis, or a history of organic neurological brain disorders and medication known to affect PPI, such as dopamine receptor antagonists. All participants gave their written consent to take part in the study. The project was approved by the Ethics Committee of the General Teaching Hospital in Prague, approval number: 37/19 Grant AZV VES 2020 1. LF UK.

Individuals with fibromyalgia were recruited directly by a rheumatologist (co-authors J.Z., L.H., L.Š.) from the Institute of Rheumatology in Prague (*n* = 6) or through an online support group for patients with fibromyalgia (*n* = 29). All individuals recruited online had rheumatological assessment and provided a medical report. The diagnosis of fibromyalgia in all fibromyalgia patients was also validated by a fibromyalgia diagnostic questionnaire.^17^

FMD patients were recruited at the specialized outpatient service for FMD at the Neurology Department, 1st Faculty of Medicine and General University Hospital in Prague to match fibromyalgia patients by age and sex. Two groups of FMD patients were recruited with and without comorbid fibromyalgia, which was diagnosed using the current criteria based on a fibromyalgia diagnostic questionnaire.^17^

Control subjects matching the FMD and fibromyalgia patients for age and sex were identified in a directory of healthy individuals willing to participate in clinical studies operated at the neurology department. They underwent a thorough screening process, including a complete medical history and a full neurological examination, ensuring that none had sensorimotor symptoms or objective signs of neurological disorders. All control subjects received financial compensation of $25.

All subjects underwent a full neurological assessment including a detailed clinical interview and examination by a neurologist with expertise in FMD (co-authors T.Se., L.N.), focusing on positive signs of functional weakness or abnormal movements that were inconsistent and incongruent with known movement disorders. FMD diagnosis was established using the Gupta and Lang criteria for a clinically definite FMD.^19^ In each individual diagnosed with FMD, we evaluated and categorized motor symptoms phenomenologically as functional weakness, tremor, dystonia/spasm, myoclonus, gait abnormalities or speech difficulties. We noted the primary motor symptom type along with any additional motor symptoms exhibited.

Assessment of fibromyalgia was performed using the 2016 Fibromyalgia Survey Questionnaire, which enabled the diagnosis of fibromyalgia based on the 2016 revised American College of Rheumatology diagnostic criteria.^17^ To meet the criteria for fibromyalgia diagnosis, the individual must have a Widespread Pain Index (WPI) of 7 or higher and a Symptom Severity Scale (SSS) score of 5 or higher. Alternatively, if the WPI is between 4 and 6, the SSS score must be 9 or higher. The individual must also have generalized pain, defined as pain in at least four of five regions, and symptoms must have been generally present for at least 3 months. The SSS includes the following symptoms: fatigue, waking unrefreshed, cognitive symptoms, headaches, pain or cramps in the lower abdomen, and depression.^17^ The WPI and SSS scores can also be combined to provide a Fibromyalgia Severity Score (FSS), which is used as a continuous measure of fibromyalgianess ranging between 0 and 31.

Individuals with fibromyalgia, FMD and fibromyalgia, FMD alone and HC were age- and sex-matched. All participants included in the study completed questionnaires for depression, anxiety and subjective cognitive complaints. Depressive symptoms were assessed using the Beck Depression Inventory (BDI-II), which consists of 21 items with a total score range of 0 to 63.^20^ Anxiety levels were assessed using the State-Trait Anxiety Inventory (STAI) trait scale, specifically the STAI X-2: a 20-item measure of trait anxiety with a possible score range of 20 to 80.^21^ Motor disorder severity was assessed using the Simplified FMD Rating Scale (S-FMDRS), which rates abnormal movements across seven body regions based on the severity and duration of symptoms, with a maximum possible score of 54.^22^ Furthermore, all subjects’ pharmacological history (selective serotonin reuptake inhibitors, serotonin–norepinephrine reuptake inhibitors, serotonin antagonist and reuptake inhibitors, noradrenergic and specific serotonergic antidepressants, tricyclic antidepressants, anticonvulsants, dopaminergic agents, benzodiazepines, opioids, non-steroidal anti-inflammatory drugs, medical cannabis) was recorded in detail (Supplementary Table 1). None of the subjects used medication known to affect PPI, such as dopamine receptor antagonists.^23-25^

A structured interview was conducted to identify medical comorbidities, family history, current medications (including hormonal contraceptives), drug use, smoking habits, caffeine consumption and handedness. Participants were asked to refrain from caffeine and smoking for 3–4 h prior to PPI testing to minimize known acute effects of these substances on sensorimotor gating and reduce interindividual variability.^6,26-28^

### Neurophysiological examination

The neurophysiological examination was conducted under standard conditions in a moderately lit and quiet room. The subject was seated comfortably, ensuring that the electromyographic device was not within their line of sight to prevent anticipation of the stimuli delivery. The subject was informed in advance about the different types of stimuli. The recording was performed using a routinely employed electrodiagnostic device (Synergy, CareFusion). The band-pass filters were set to frequencies ranging from 30 Hz to 30 000 Hz, with a sampling frequency of 2000 Hz.

#### Paradigm

The examination involved electromyographic recording of muscle activity in the orbicularis oculi using 10 mm gold surface electrodes with conductive gel. The active electrode was placed along the midline under the eye, dividing the muscle into two symmetrical parts, while the reference electrode was positioned 2 cm laterally toward the outer corner of each eye. To elicit the blink reflex, a 0.5 ms rectangular pulse of constant current was delivered to the right supraorbital nerve. The cathode was placed over the supraorbital incisura, and the anode was positioned 3 cm laterally along the nerve’s path over the eyebrow.

The stimulation intensity applied was 10 times the individual’s sensory threshold, which is defined as the smallest stimulus intensity perceived by the subject in at least 4 of 8 applications. A prepulse stimulus, consisting of a 0.2 ms rectangular pulse of constant current, was applied 100 ms before the supraorbital nerve stimulation, using ring electrodes attached to the right index finger over the proximal and medial phalanges. A 100 ms interval between the prepulse and the startle stimulus was used to ensure reliable inhibition of the blink reflex and consistency with previous PPI studies of the blink reflex in FMD and fibromyalgia.^5,6,29^ The prepulse stimulation intensity was set at twice the subject’s sensory threshold. Each subject underwent nine stimulations of the supraorbital nerve alone (baseline blink reflex) and nine stimulations with a prepulse preceding the supraorbital nerve stimulation (blink reflex with prepulse), with approximately 8–10 s pauses between individual stimulations.

During the baseline trials and prepulse trial, the morphology of blink reflex (R1, R2 and R3 components) was carefully assessed, and only trials from individuals with the R1 component demonstrating no change or increase in the prepulse trials were included in the study. The examination also involved assessing the subject’s perceived discomfort or pain using the Numerical Rating Scale for Pain (NRS), where 0 indicates no discomfort and 10 represents unbearable pain.

### Analysis

Electromyographic recordings were rectified and analysed offline. Each subject underwent 18 stimulations (nine for the blink reflex and nine for the blink reflex with prepulse). The early R1 and late R2 components were identified in each ipsilateral EMG recording of the blink reflex, with the R2 component also recorded contralaterally (R2c). The magnitude of the R2 and R2c blink reflexes was measured as the area under the curve (ms/mV) and averaged. In some recordings, an R3 component, a late polyphasic part of the blink reflex, was also observed but was not included in the analysis.

To evaluate PPI, we calculated the average blink reflex magnitude (R2 and R2c areas) for each trial. For each individual, we averaged the values from nine trials per condition (baseline and prepulse).

To normalize data among subjects, we expressed the change in blink reflex magnitude during prepulse trials relative to baseline trials as a percentage of the baseline trials (%PPI = mean blink reflex magnitude in prepulse trials/mean blink reflex magnitude in baseline trials × 100). The primary outcome, PPI size, was calculated as the difference in blink reflex magnitude between the prepulse (%PPI) and baseline trials (100%), i.e. PPI size = 100% − %PPI. The PPI size value represents the primary outcome and reflects the degree of prepulse inhibition in a given subject. Blink reflex recordings from all participants were of sufficient quality to allow for further analysis.

### Statistics

A multiple linear regression model of FSS as a measure of fibromyalgianess was fitted with PPI as the predictor, adjusting for age and sex to account for potential confounding effects. Additionally, we also fitted similar models of WPI and SSS. Because depression and anxiety are strongly associated with chronic pain^30^ and may act as mediators of the relationship between PPI size and fibromyalgia symptoms, we did not include them as covariates in these models. We also examined whether PPI size was associated with motor symptom severity in a subset of patients with FMD, including those with comorbid fibromyalgia (*n* = 70), to assess whether reduced PPI size is specific to pain symptoms within FMD or also relates to motor symptom severity.

Additionally, group differences in demographic and clinical characteristics (age, illness duration, FSS, WPI, SSS, S-FMDRS, BDI-II and STAI-X2) were assessed using analysis of variance with Tukey *post hoc* tests. Pearson’s correlation was used to assess the relationship between PPI size and other symptom measures, including the BDI-II, STAI-X2 and S-FMDRS. Analyses were performed in R (version 4.4).^31^  *P*-values arising from multiple statistical testing were corrected using the Holm–Bonferroni method.^32^

### Ethical compliance statement

The study was approved by the local Ethics Committee of the General University Hospital in Prague (Approval Nr. 37/19) and all participants gave their written consent to take part in the study. We confirm that the work is consistent with the *Brain* guidelines on ethical publication.

## Results

A total of 140 participants were included in the analysis, comprising 35 subjects per patient group and 35 healthy controls. All patient cohorts and healthy controls were matched for key demographics with no differences in age or sex among the groups. Demographic data and clinical scores are summarized in Supplementary Table 2. *Post hoc* tests showed that the illness duration was significantly longer in fibromyalgia patients as compared with FMD with fibromyalgia (*P* = 0.01) and FMD patients (*P* < 0.001), but the illness duration between FMD and FMD with fibromyalgia did not differ (*P* = 0.61). Healthy controls reported significantly lower FSS, WPI, SSS, BDI-II and STAI-X2 scores compared with all patient cohorts (*P* < 0.001). Fibromyalgia only and FMD with fibromyalgia patients had significantly higher FSS, WPI, SSS scores (*P* < 0.001), BDI-II and STAI-X2 scores (*P* < 0.01) compared with the FMD group. S-FMDRS was significantly higher in FMD with fibromyalgia patients compared with FMD only patients (*P* < 0.001). There were no significant differences in the clinical scales between fibromyalgia only and FMD with fibromyalgia patients.

In the full sample of all patients and healthy controls, three multiple linear regression models were conducted to examine whether fibromyalgia-related symptom severity, as measured by the FSS, WPI and SSS is predicted by PPI size, age and sex. The regression model for FSS explained 22.5% of the variance, *F*(3,136) = 14.43, *P* < 0.001. The models for WPI and SSS explained 19.6% and 19.9% of the variance, respectively [WPI: *F*(3,136) = 12.26, *P* < 0.001; SSS: *F*(3,136) = 12.52, *P* < 0.001].

Across all three models, PPI size was a significant negative predictor after adjusting for sex and age, indicating that lower PPI was associated with greater fibromyalgia-related symptom severity. Full regression coefficients are presented in Table 1.

**Table 1:** Regression coefficients for predictors of fibromyalgia-related scales

Predictor	Outcome		
	FSS (SE)	WPI (SE)	SSS (SE)
Intercept	30.26 (4.74)***	18.39 (3.17)***	11.87 (1.96)***
PPI size	−0.21 (0.03)***	−0.13 (0.02)***	−0.08 (0.01)***
Age	−0.11 (0.08)	−0.08 (0.05)	−0.03 (0.03)
Sex	−3.68 (2.31)	−2.49 (1.54)	−1.19 (0.95)

Regression coefficients are reported. FSS = Fibromyalgia Severity Scale; PPI = Prepulse Inhibition; SE = standard error; SSS = Symptom Severity Scale; WPI = Widespread Pain Index. ***P < 0.001.

A multiple linear regression was conducted to examine whether PPI size, age and sex predicted motor symptom severity, as measured by the S-FMDRS, in a subset of patients with FMD (*n* = 70). The model did not explain the S-FMDRS scores in a significant way, *F*(3,66) = 1.50, *P* = 0.22, with an adjusted *R²* of 0.02. PPI size was not a significant predictor [*t*(66) = −0.33, *P* = 0.75], nor were age [*t*(66) = 1.82, *P* = 0.066] or sex [*t*(66) = −0.80, *P* = 0.42]. These findings suggest that PPI size is not associated with motor symptom severity in this patient subgroup.

To further examine the specificity of the relationship between PPI size and fibromyalgia symptoms, we conducted a multiple regression analysis predicting FSS from PPI size, adjusting for the effects of S-FMDRS scores, age and sex. The model accounted for 14.7% of the variance in fibromyalgia symptom severity, *F*(4,65) = 3.97, *P* = 0.006. PPI size remained a significant negative predictor of fibromyalgia symptoms [−0.10, *t*(65) = −2.58, *P* = 0.019], even after adjusting for motor symptom severity. S-FMDRS was also a significant predictor [0.25, *t*(65) = 2.55, *P* = 0.016], while age and sex were not associated with fibromyalgia severity. These results suggest that reduced PPI size is specifically associated with pain-related symptoms in FMD, independent of motor symptom burden.

**Figure 2:**
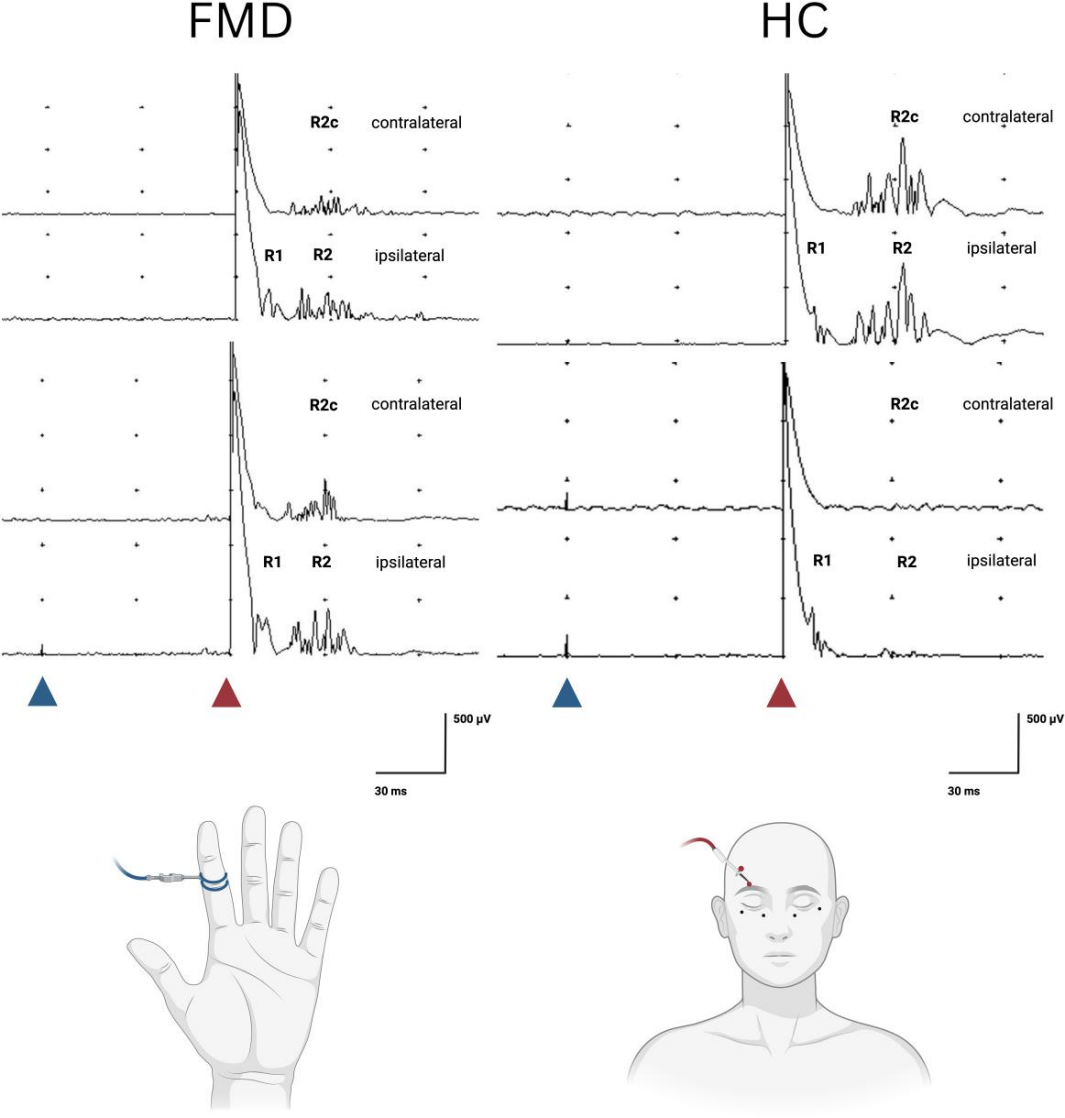
**Blink reflex recordings in a patient with functional motor disorder and a healthy control.** A representative example of the blink reflex recordings elicited by the electrical supraorbital nerve stimulation (pulse) without (*top two traces*) and with prepulse stimulation (*bottom two traces*) in an functional motor disorder (FMD) patient (*left*) and a control (HC) subject (*right*). Blue arrowhead indicates prepulse stimulus to the index finger. Red arrowhead indicates supraorbital nerve stimulation. Note that the suppression of the R2 and R2c area under the curve was lower in the patient than in the control. Created in BioRender. Nováková, L. (2025) https://BioRender.com/oya9ami.

Examples of blink reflex responses without and with prepulse stimulation in a patient and a healthy control subject are shown in Fig. 2.

### Additional analyses

**Figure 3:**
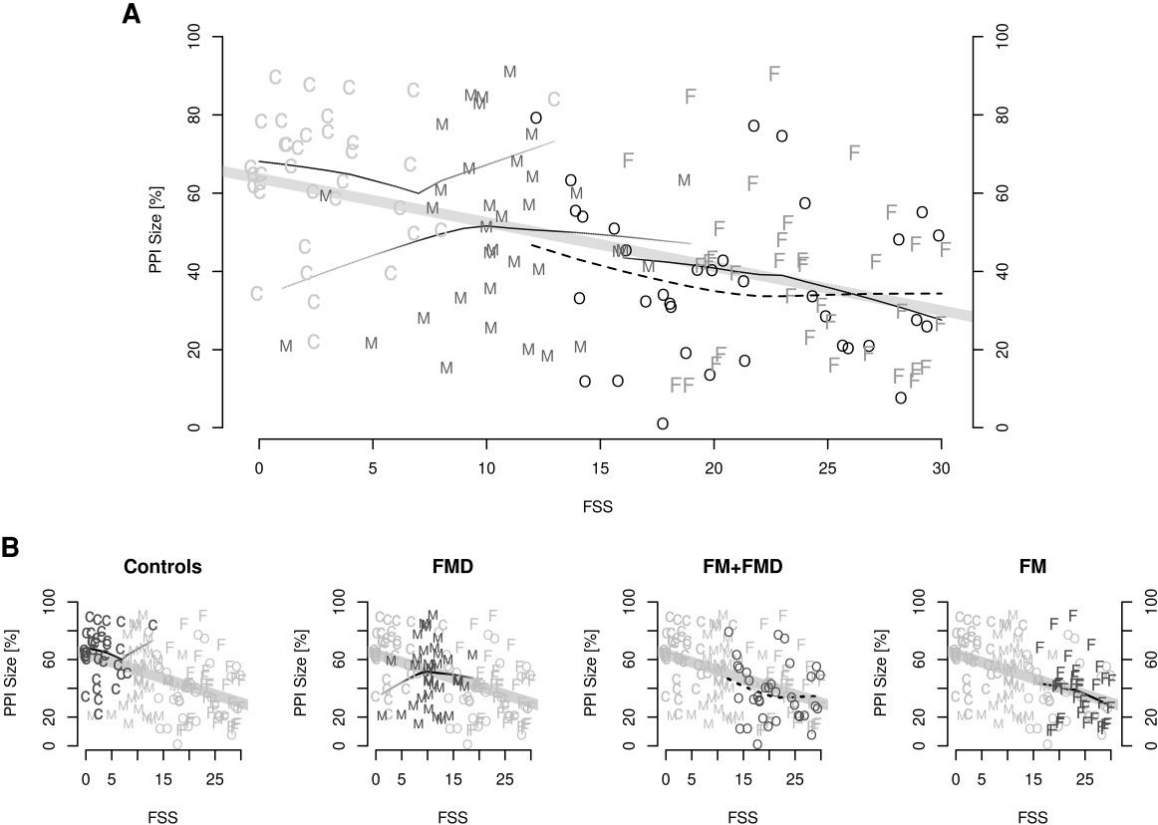
**The relation between fibromyalgianess and prepulse inhibition.** On average, a decrease of prepulse inhibition (PPI size) by 10%, was linked to an increase of fibromyalgianess (FSS) by 2 in a model of FSS explained in terms of PPI size, age and sex (grey line). FSS values were slightly jittered to ease visual perception of overlapping values. C = healthy controls; F = fibromyalgia; M = functional motor disorder; O = functional motor disorder and fibromyalgia; FSS = Fibromyalgia Severity Scale; PPI size = prepulse inhibition size (i.e. the difference between the mean blink reflex magnitude in the baseline trials and the trials with the prepulse expressed in %).

Correlation analysis (Fig. 3 and Supplementary Fig. 1) revealed a negative association between PPI size and fibromyalgia-related measures (FSS, WPI, SSS) and affective symptoms (BDI-II, STAI-X2), but no correlation with motor symptom severity (S-FMDRS). To further support the view of fibromyalgianess as a continuum, the ANOVA model showed that between-group differences in PPI size (Supplementary Fig. 2A) disappeared after adjustment for fibromyalgia severity (FSS) (Supplementary Fig. 2B), indicating that these differences were artefacts of the strong negative correlation with fibromyalgia-related scales and artificial diagnostic cut-offs.

## Discussion

In this study, we analysed PPI size as a measure of subcortical early processing and integration of sensory information^33^ across individuals with two conditions with partially overlapping symptoms, FMD and fibromyalgia. Reduced PPI size was associated with higher scores of fibromyalgianess across all participants. In FMD, comorbid fibromyalgia was associated with more severe motor symptoms; however, clinician-rated motor symptom severity was not predicted by PPI size. These findings suggest that PPI size is a relevant physiological marker of fibromyalgianess, rather than motor symptom severity.

**Figure 4:**
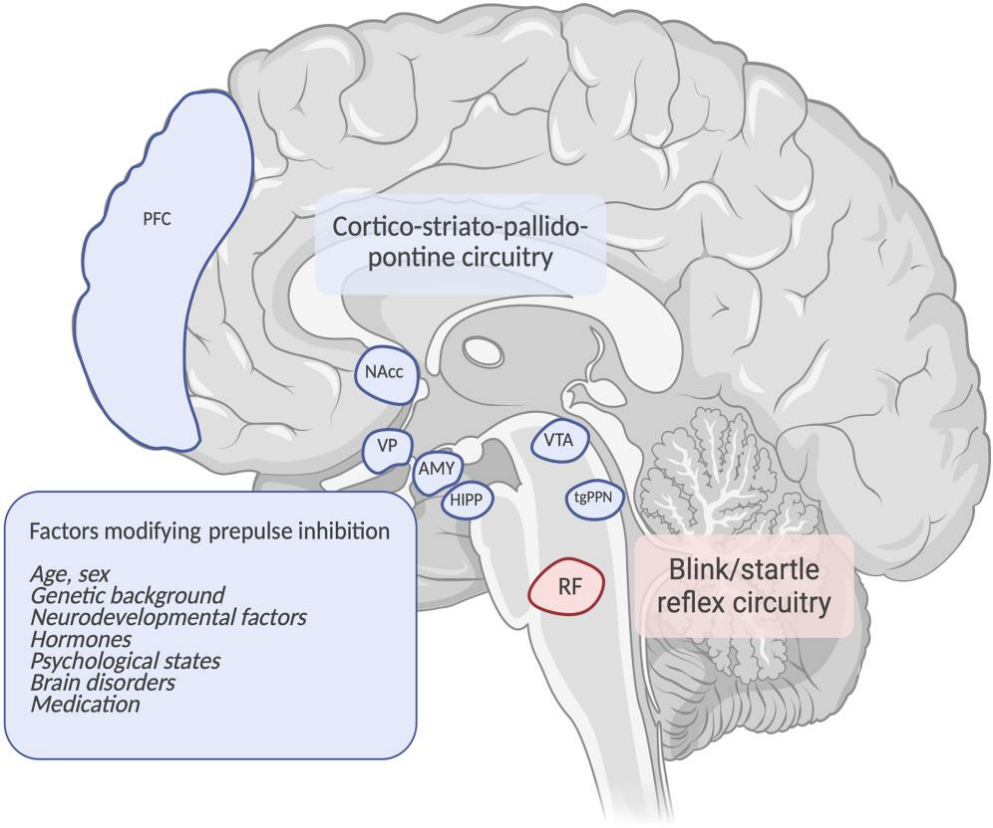
**Neural circuitry and contributing factors influencing prepulse inhibition.** Top-down regulatory mechanisms that mediate prepulse inhibition involve projections from forebrain structures. This cortico-striato-pallido-pontine circuitry involving the prefrontal cortex (PFC), the nucleus accumbens (NAc), the ventral pallidum (VP), the amygdala (AMY), the hippocampus (HIPP) and the ventral tegmental area (VTA) projects via the tegmental pedunculopontine nucleus (TgPPN) to the pontine blink reflex/startle response circuitry involving pontomedullary reticular formation.^38^ The regulatory control of prepulse inhibition mediated by the forebrain is influenced by various physiological and pathophysiological factors (see box), which affect attention re-orienting and stimulus-related salience attribution processes, both of which have been implicated in functional neurological disorder.^33^ Created in BioRender. Nováková, L. (2025) https://BioRender.com/v55wx1a.

Extensive research has identified the neuroanatomical and neurochemical substrates of PPI, particularly within the forebrain and pontine tegmentum, with the ventral striatum playing a central role in this regulatory network.^33-37^ PPI reflects early, pre-attentive filtering of sensory information, protecting ongoing stimulus processing from disruption.^38^ PPI size is influenced by stimulus salience and reflects automatic selection processes that occur before conscious awareness.^29,39-42^ Without intact PPI, low-salience stimuli may fail to be appropriately integrated with or filtered against competing high-salience inputs, resulting in decreased sensory fidelity and a noisier perceptual environment for subsequent processing. Disruptions in PPI have been observed in a range of (but not all) neuropsychiatric disorders,^33,43^ including both motor and seizure subtypes of FND,^4,5^ as well as some somatic symptom disorders with prominent pain.^6-8^ Abnormal PPI has been associated with different pathophysiological mechanisms reflecting diverse underlying mechanisms, including genetic vulnerability and neural circuit dysfunction (Fig. 4).^33,44^ As such, PPI is increasingly recognized as a transdiagnostic marker of impaired sensorimotor gating in psychopathology.

Here, we extended previous findings of PPI deficits from small sample studies comparing healthy controls with a single clinical group with FMD or fibromyalgia.^6,9^ In contrast to the previous fibromyalgia study (*n* = 10),^6^ we used current diagnostic criteria for fibromyalgia which are based on self-reported widespread pain and the severity of other somatic and psychological symptoms (i.e. fibromyalgianess) using predefined cut-offs.^17^ However, a varying degree of fibromyalgianess exists in healthy subjects and various clinical populations. It has been pointed out that reported neurobiological and imaging abnormalities in fibromyalgia may largely reflect sampling bias from dichotomizing patients and selecting only extremes of symptom severity distribution.^18^ In line with these observations, the between-group differences in PPI size found in this study disappeared after adjustment for FSS, indicating that the apparent effects were an artefact of the correlation between PPI and fibromyalgia severity and categorical classification based on diagnostic cut-offs.

In this study, using fibromyalgianess as the outcome measure allowed us to move beyond categorical diagnoses with arbitrary cut-off values and instead examine illness features across four matched groups, including individuals with overlapping conditions and healthy controls. The clinical correlates of PPI deficits across clinical populations remain poorly understood. For the first time, we report an association between PPI size and fibromyalgianess across all groups of subjects, including healthy controls. This finding aligns with prior literature on reduced PPI in different chronic pain conditions such as fibromyalgia, interstitial cystitis and irritable bowel syndrome,^6-8^ but also findings in migraine patients showing that PPI reduction is more likely in those with allodynia.^45^ In a separate analysis of patients with FMD (with and without fibromyalgia), we found that PPI size was associated with fibromyalgianess, but not with clinician-rated motor symptom severity. These findings support the interpretation that reduced PPI is associated with perceived non-motor symptom severity in FND, i.e. subjective experience of fibromyalgianess. Indeed, self-reported symptom severity measures are strongly intercorrelated in FMD in this and other studies, likely reflecting a perceptual impairment across multiple symptom domains.^9^ Future research should explore how PPI interacts with other biological, psychological and social factors across diagnostic boundaries to better understand symptom development and maintenance.

A key principle of the current neurobiological model based on predictive coding account is that the same neural/computational mechanism can account for all functional symptoms including pain, regardless of specific phenotype.^46-48^ This model highlights the role of abnormal (symptom specific), overly precise expectations that overwhelm incoming sensory data which would indicate the absence of the specific symptom. However, the results from this study suggest that different symptoms (motor versus fibromyalgianess) coexisting within one individual do not share the same neurophysiological signature. PPI seems to be a specific transdiagnostic biomarker of fibromyalgianess. We can hypothesize that while PPI size is linked to symptom perception/experience, the motor symptoms could be more directly associated with motor outputs, involving distinct underlying circuits and functional anatomy. Abnormal top-down regulatory mechanisms that mediate PPI, involving projections from forebrain structures to the pontine reflex circuitry, are likely the primary network responsible for impaired PPI in fibromyalgia and in FMD in which it seems to be associated with non-motor symptom severity including pain.

In patients with FND and fibromyalgia, functional imaging studies have shown dysfunction in the salience network, including the ventral striatum and amygdala, and in the ventral attention network.^14,49-57^ These networks are crucial for detecting salient stimuli and reorienting focus, as well as integrating contextual information through bottom-up and top-down interactions and attentional shifts.^40,58,59^ Abnormal processing of physiologically salient stimuli and disrupted attentional shifts in FND could be related to PPI impairment in this disorder. There is evidence in FMD of abnormalities in sensory processing, which would be consistent with an abnormality in sensory integration, resulting in noisy early stages of sensory processing.^29^ This was the conclusion of previous work showing abnormalities in temporal discrimination in people with motor subtype of FND, where data analysis using drift diffusion modelling suggested abnormalities in evidence accumulation.^15,55^ There is also evidence for impaired processing of interoceptive information in both FND and fibromyalgia.^56,57^ This raises the possibility that impaired PPI could reflect both a trait abnormality that makes people more vulnerable to developing FND, fibromyalgia or other functional disorders, and a marker of the current state of downregulation of sensory input in established illness.

The association between PPI size and fibromyalgianess across all groups of subjects is an important finding, as PPI is considered to be a stable neurophysiological marker in healthy individuals.^60,61^ In schizophrenia, a higher heritability of reduced PPI was found in families with a strong genetic predisposition, suggesting that PPI deficits may indicate genetic vulnerability.^62^ This vulnerability could potentially be associated with other neuropsychiatric disorders including FND, and their common comorbidities affecting PPI such as obsessive-compulsive disorder, generalized anxiety disorder and post-traumatic stress disorder (PTSD).^63^ For example, a longitudinal study of 1226 US Marines and Navy Corpsmen found that individuals in the top 25% of PPI had over a 50% reduced likelihood of developing PTSD after deployment (odds ratio = 0.32), suggesting strong sensorimotor gating may protect against PTSD.^63^ Abnormal PPI could also indicate a link between genetic risk variants and more direct lower-level biological processes contributing to observable symptoms. This would be consistent both with known risk factors for FND and fibromyalgia, such as traumatic experiences, depression, anxiety, neurodiversity and joint hypermobility, which are all characterized by abnormalities in interoceptive and more general sensory processing and also impaired PPI in their own right.^64,65^ There is evidence suggesting that noisy information is more prone to be overridden by top-down expectations, such as the less precise visceral input mediated by the autonomous nervous system, i.e. interoceptive information.^66^ We can speculate that if abnormal expectations are formed in a brain that is unable to efficiently filter the incoming sensory signals, the expectations are more likely to overwhelm such noisy inputs and result in development and maintenance of functional symptoms.^12^ Our findings thereby challenge the central sensitization hypothesis still prevalent in fibromyalgia literature,^12^ which assumes heightened sensitivity to peripheral input; however, there is a lack of neurophysiological correlates to behavioural readouts of chronic pain in studies on central sensitization in humans.^67^ Instead, our results imply that the system is less sensitive to peripheral input, leaving perception disproportionately shaped by top-down predictions.

In this study, we have linked an objective neurophysiological marker to subjectively reported fibromyalgianess, which has been found to be a useful prognostic factor for outcomes for several types of interventions, including pain following hip surgery or persistent pelvic pain after hysterectomy.^68,69^ Higher scores have been associated with worse outcomes, even in individuals who do not meet the full diagnostic criteria for fibromyalgia.^70^ So far, objective markers of widespread pain are limited/lacking. Further studies should address the utility of PPI size as an objective measure of widespread pain and it’s association with clinical outcomes for people undergoing surgical procedures for chronic pain.^71^ Further studies using multimodal approaches are needed to identify specific neurophysiological profiles, including other neurophysiological measures such as assessment of cortical inhibitory mechanisms previously reported to be abnormal in FMD^72,73^ or combinations of neurophysiological and laboratory and neuroimaging pain markers.

Moreover, current and previous studies have found normal PPI in some individuals with these disorders and reduced levels in some healthy individuals. This could reflect the limitations of diagnostic classifications, as clinical criteria, though established through expert consensus, are somewhat arbitrary. Research indicates that individuals who meet the diagnostic criteria for a disorder and those who narrowly miss the cut-off often share significant similarities, differing mainly in the number and severity of symptoms. Therefore, examining the impact of comorbidities, cognitive measures and therapeutic interventions, along with deep phenotyping and identifying FND and fibromyalgia subtypes, could uncover neurophysiological mechanisms and advance neurotherapeutic development addressing core dysfunctions rather than disorder-specific symptoms. Other factors, such as sleep disturbance, which is known to affect PPI and is prevalent in both fibromyalgia and FMD,^74-77^ was not specifically assessed in this study; future research should examine its potential contribution to altered sensorimotor gating in these populations. PPI shows promise as a transdiagnostic endophenotype for genomic studies and a biomarker for healthy brain circuitry, which may predict sensitivity to psychotherapeutics.^25,33^ Future research could explore whether the associations found in this study exist in other populations, such as schizophrenia. This would also help clarify whether PPI can serve as a transdiagnostic marker measuring somatosensory integration or early attentional processing mechanisms related to symptom perception in different populations rather than being a generalized index of disease presence.

A single marker is unlikely to sufficiently explain such complex and overlapping disorders, so these findings highlight the need for future research on combined markers. Previous pain research has identified other promising markers.^78^ For example, laser evoked potentials have shown correlation of amplitude with nociceptive stimulation intensity suggestive of top-down processing impairment; EEG studies have identified correlations between pain and the power at different EEG frequency bands; other potential correlates of chronic pain include laboratory markers such as beta endorphine, calcitonin gene-related peptide, vasoactive intestinal peptide, substance P, endocannabinoids (AEA and 2-AG) and serum-derived soluble intercellular adhesion molecule1, and a specific neuroimaging pattern correlating with fibromyalgianess has been identified.^78^ It may be, like PPI, that such markers are not specific to fibromyalgia or even to the presence of widespread pain but might also occur in people with FND without fibromyalgia.

In conclusion, we found a deficit of PPI that suggests abnormal early processing of somatosensory inputs in FND and fibromyalgia. A lower PPI size was associated with more fibromyalgianess, i.e. pain widespreadness and higher levels of non-motor symptoms in people with fibromyalgia and FMD. PPI thus could represent a valuable objective marker of generalized chronic pain and associated non-motor symptoms in this clinical population.

## Supplementary Material

awaf437_Supplementary_Data

## Data Availability

The data that support the findings of this study are available on request from the corresponding author. All data will be anonymized.
