# Supplementary material for: Prepulse inhibition of the blink reflex in functional neurological disorder and fibromyalgia
Source: Brain. 2025 Nov 21;149(7):2323–32. doi: 10.1093/brain/awaf437 (PMC13337237; doi:10.1093/brain/awaf437)
Supplement: awaf437_Supplementary_Data [file awaf437_supplementary_data.pdf]

Supplementary material

Methods

Table S1 Medication use

	FM (n=35)	FMD+FM (n=35)	FMD (n=35)	HC (n=35)
Selective Serotonin Reuptake Inhibitors	11	8	10	5
Serotonin-Norepinephrine Reuptake Inhibitors	7	5	1	1
Serotonin Antagonist and Reuptake Inhibitors	9	7	5	1
Noradrenergic and Specific Serotonergic AD	2	3	0	0
Tricyclic AD	2	0	0	0
Anticonvulsants	2	1	0	0
Dopaminergic Agents	4	2	2	0
Benzodiazepines	6	5	2	1
Opioids	10	5	1	0
Nonsteroidal Anti-inflammatory Drugs	22	21	6	1
Medical Cannabis	8	2	0	0
Pregabalin/Gabapentin	12	21	8	0

Abbreviations: AD = antidepressants; FM = fibromyalgia; FMD = functional motor disorder; FMD+FM = functional motor disorder and fibromyalgia ; HC = Healthy Controls

Results

Demographic and clinical data

Table S2 Demographic and clinical measures

	**FMD+FM** (n=35)	**FM** (n=35)	**FMD** (n=35)	**HC** (n=35)	**Test statistics**	***P-value***	**Effect size**
**Age**	48.6 (8.3)	50.1 (7.8)	47.3 (13.4)	48.3 (8.3)	F(3, 136) = 0.29	0.83	0.006 [a]
**Sex (F/M)**	32/3	32/3	32/3	32/3	NA	NA	NA
**Duration (y)**	7.40 (6.30)	12.50 (9.40)	5.70 (5.40)	NA	F(2, 102) = 8.35	<0.001	0.14 [a]
**FSS**	20.82 (5.31)	23.89 (3.90)	10.51 (3.60)	2.86 (2.94)	F(3, 136) = 200.59	<0.001	0.82 [a]
**WPI**	12.09 (4.34)	14.20 (2.94)	4.28 (2.46)	1.26 (1.48)	F(3, 136) = 150.22	<0.001	0.77 [a]
**SSS**	8.74 (2.05)	9.69 (1.64)	6.26 (2.79)	1.60 (1.91)	F(3, 136) = 101.95	<0.001	0.69 [a]
**S-FMDRS**	15.17 (8.02)	NA	9.71 (5.59)	NA	t(68) = 3.85	<0.002	0.92 [b]
**BDI-II**	24.23 (11.26)	27.71 (13.00)	17.20 (11.63)	6.03 (6.71)	F(3, 136) = 27.07	<0.001	0.38 [a]
**STAI-X2**	49.94 (12.83)	50.80 (12.27)	44.20 (11.92)	33.23 (6.64)	F(3, 136) = 18.70	<0.001	0.29 [a]

Mean values (SD) are presented; P-values are nominal values corrected using the Holm-Bonferroni method.

Abbreviations: BDI-II = Beck Depression Inventory-II; FM = Fibromyalgia; FMD = Functional Motor Disorder; FMD+FM = Functional Motor Disorder and Fibromyalgia; Fibromyalgia Severity Scale; HC = Healthy controls; S-FMDRS = Simplified Functional Movement Disorders Rating Scale; SSS = Symptom Severity Scale; STAI-X2 = State-Trait Anxiety Inventory – trait; WPI = Widespread Pain Index; [a]Partial η^2; [b]Cohen's d.

Note: Disease duration represents FMD duration in patients with FMD (with or without FM) and FM duration in patients with FM alone.

Correlation analyses

In all subjects (n=140), prepulse inhibition size (PPI size) was negatively correlated with all fibromyalgia-related measures (i.e. FSS, WPI, SSS), as well as with depression (BDI-II) and anxiety (STAI-X2) scores. All fibromyalgia-related scales were positively correlated with both BDI-II and STAI-X2. In the subgroup comprising patients with FMD (n=70), motor symptom severity (S-FMDRS) was not correlated with PPI size. Of the fibromyalgia-related measures, the FSS and SSS were significantly correlated with S-FMDRS. Moreover, S-FMDRS was positively correlated with both BDI-II and STAI-X2. See Figure S1 for further details.

Fig S1. Cross-correlations between prepulse inhibition size, fibromyalgia-related measures, affective symptoms, and motor symptom severity.

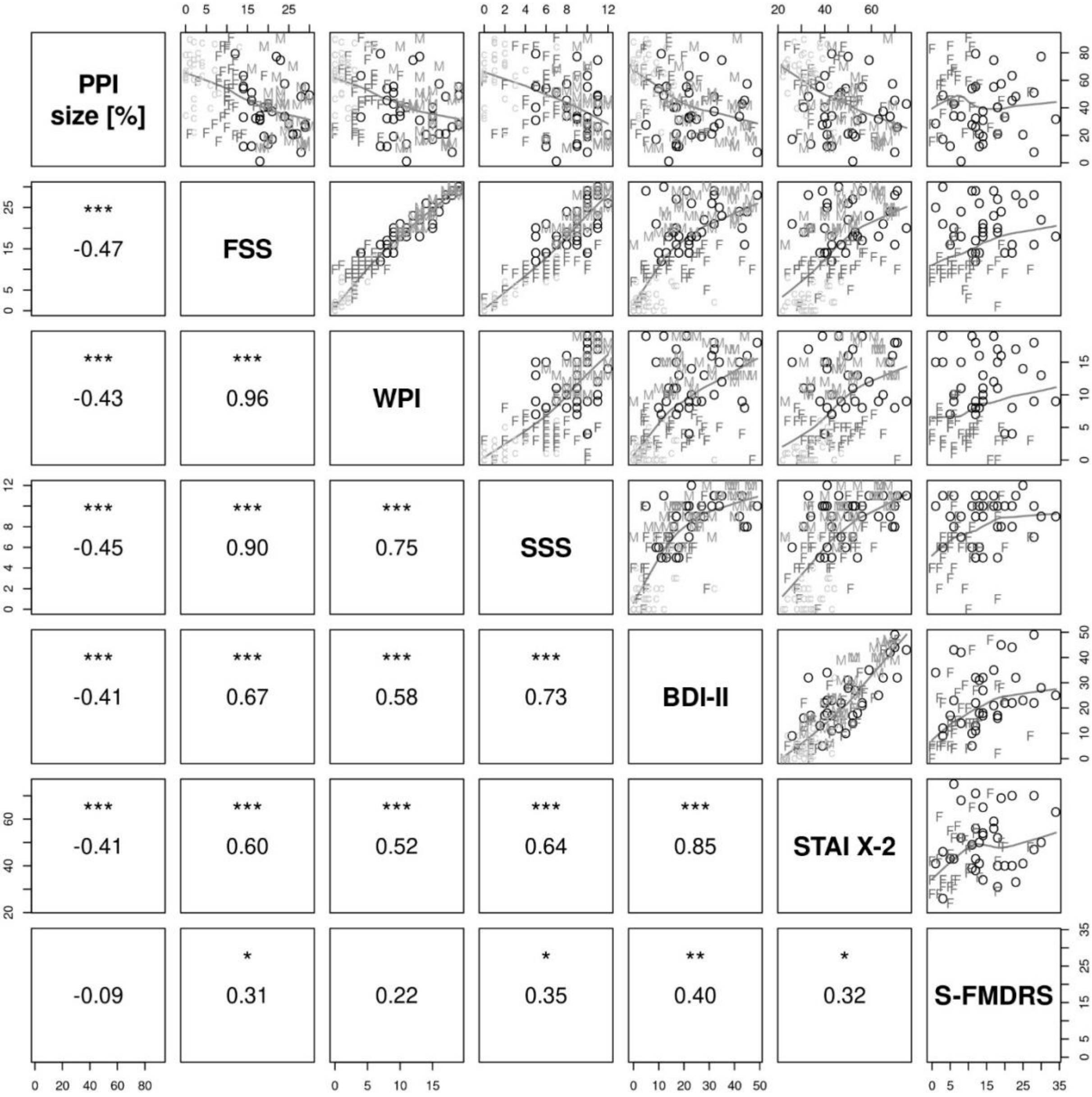

Pearson correlation coefficients are shown; *** $p < 0.001$, ** $p < 0.01$, * $p < 0.05$.

Abbreviations: BDI-II = Beck Depression Inventory-II; C = healthy controls; F = patients with fibromyalgia; FSS = Fibromyalgia Severity Scale; M = patients with functional motor disorder; O = patients with functional motor disorder and fibromyalgia; PPI size = Prepulse Inhibition size; S-FMDRS = Simplified Functional Movement Disorder Rating Scale; SSS = Symptom Severity Scale; STAI-X2 = State-Trait Anxiety Inventory trait; WPI = Widespread Pain Index.

Between-group comparison of prepulse inhibition size

PPI size differed across the four groups as revealed by the ANOVA model with a significant effect of the group on the PPI size, $F(3,136) = 15.47$, $p < 0.001$, with a large effect size, $\eta^2 = 0.25$ (Fig. S2 A). The Tukey post-hoc test indicated that the HC generally had a higher PPI size compared to all patient groups. The FMD with fibromyalgia had significantly lower PPI size than the FMD only group. There were no significant differences in PPI size between the FMD with fibromyalgia and fibromyalgia only groups nor between the FMD only and fibromyalgia only. However, when we adjusted the PPI size for FSS (as PPI size was strongly negatively correlated with FSS), PPI size no longer differed across the four groups, $F(3,136) = 0.87$, $p = 0.46$ (Fig. S2 B).

Fig S2. Between-group comparison of prepulse inhibition size

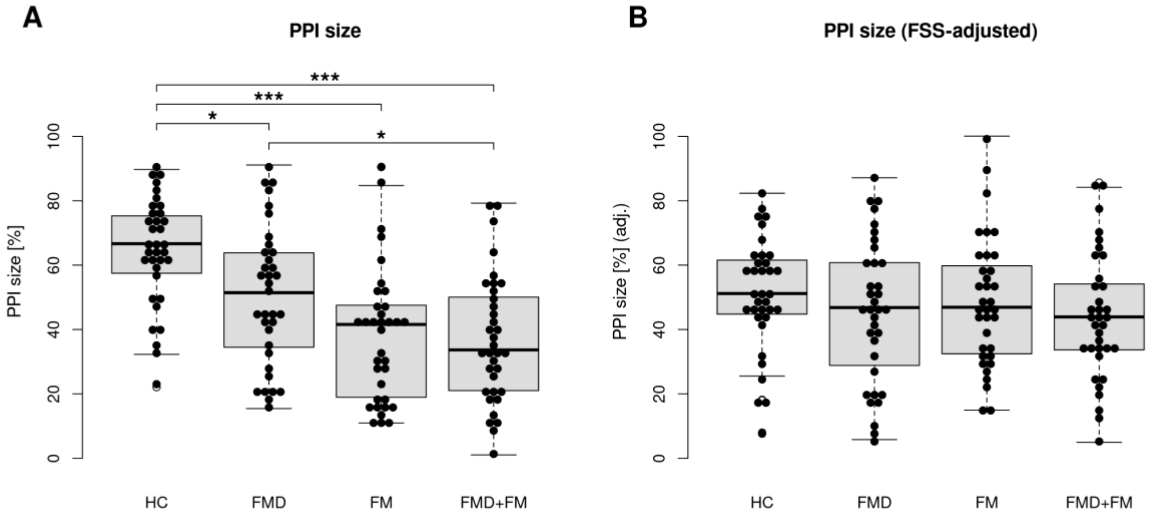

Prepulse inhibition size (PPI size) in patients with fibromyalgia alone (FM), with functional motor disorder and fibromyalgia (FMD+FM), with functional motor disorder alone (FMD), and healthy controls (HC). The PPI size (i.e. the difference between the mean blink reflex magnitude in the baseline trials and the trials with the prepulse) is expressed in %. **A**: PPI size differed between groups. **B:** PPI size adjusted for FSS did not differ across groups.

*** p < 0.001, * p < 0.05.
